# Dolutegravir plus rilpivirine: benefits beyond viral suppression: DORIPEX retrospective study

**DOI:** 10.1097/MD.0000000000029252

**Published:** 2022-06-17

**Authors:** Jesús Troya, Carlos Dueñas, Idoia Irazola, Ignacio de los Santos, Sara de la Fuente, Desiré Gil, Cristina Hernández, María José Galindo, Julia Gómez, Elisabeth Delgado, Estela Moreno-García, Guillermo Posada, Teresa Aldámiz, Jose Antonio Iribarren, José Manuel Guerra, Miguel Ángel Morán, Carlos Galera, Javier Fuente, Ana Peláez, Miguel Cervero, María Garcinuño, Marta Montero, Francisco Ceballos, Luis Buzón

**Affiliations:** a Hospital Universitario Infanta Leonor, Madrid, Spain; b Hospital Clínico Universitario de Valladolid, Valladolid, Spain; c Hospital Universitario de Galdakao, Vizcaya, Spain; d Hospital Universitario la Princesa, Madrid, Spain; e Hospital Universitario Puerta de Hierro, Madrid, Spain; f Hospital Miguel Servet, Zaragoza, Spain; g Hospital Universitario Príncipe de Asturias, Madrid, Spain; h Hospital Clínico de Valencia, Valencia, Spain; i Hospital Río Hortega, Valladolid, Spain; j Hospital San Juan, Alicante, Spain; k Complejo Hospitalario de Navarra, Pamplona, Spain; l Hospital Álvaro Cunqueiro, Vigo, Spain; m Hospital Universitario Gregorio Marañón, Madrid, Spain; n Hospital Universitario de Donostia, Donostia, Spain; o Complejo Asistencial Universitario de León, León, Spain; p Hospital de Vitoria. Vitoria. Spain; q Hospital Universitario Virgen de la Arrixaca. Murcia, Spain; r Hospital Povisa Ribera de Vigo, Vigo, Spain; s Hospital Rafael Méndez. Lorca, Spain; t Hospital Universitario Severo Ochoa, Madrid, Spain; u Complejo Asistencial de Ávila, Ávila, Spain; v Hospital Universitario y Politécnico la Fe, Valencia, Spain; w Unit of Viral Infection and Immunity, National Center for Microbiology (CNM), Health Institute Carlos III (ISCIII), Majadahonda, Madrid, Spain; x Hospital de Burgos, Burgos, Spain.

**Keywords:** dolutegravir, dual therapy, HIV-1, rilpivirine, switching

## Abstract

Switching dual therapy with dolutegravir (DTG) plus rilpivirine (RPV) was assessed in the SWORD-1 and SWORD-2 studies. Real-life data regarding the immunological impact of this approach on CD4+ and CD8+ T lymphocyte counts and the CD4/CD8 ratio are scarce. We evaluated this strategy on the basis of clinical practice data.

A multicentric retrospective cohort study.

Treatment-experienced virologically suppressed HIV-1-infected patients who were switched to DTG plus RPV were included. Using different models for paired data, we evaluated the efficacy and immune status in terms of CD4+ and CD8+ T-cell counts and CD4/CD8 ratio at 24 and 48 weeks of treatment.

The study population comprised of 524 patients from 34 centers in Spain. Men accounted for 76.9% of patients, with a median age of 53 years. Patients receiving DTG plus RPV reached weeks 24 and 48 in 99.4% and 83.8% of cases, respectively, with only three (0.57%) virological failures. We found a significant decrease in CD8+ T-cell count (log OR –40) at week 24 and an increase in CD4+ T-cell count at week 48 (log OR +22.8). In acquired immunodeficiency syndrome-diagnosed patients, we found a significant increase in the CD4+ T-cell count at week 48 (log OR = 41.7, *P* = .0038), but no significant changes in the CD8+ T-cell count (log OR = –23.4, *P* = .54). No differences were found in the CD4/CD8 ratio between the acquired immunodeficiency syndrome subgroup and sex or age.

In patients with controlled treatment, dual therapy with DTG plus RPV slightly improved the immune status during the first 48 weeks after switching, not only in terms of CD4+ T-cell count but also in terms of CD8+ T-cell count, with persistently high rates of viral control.

## 1 Introduction

Antiretroviral therapy with three active drugs has been recognized as the standard of care for the treatment of human immunodeficiency virus type 1 (HIV-1) infection for the last 25 years. This therapeutic strategy, based on the combination of a backbone of 2 nucleos(t)ide reverse transcriptase inhibitors (NRTIs) and a third agent that could be a non-nucleoside reverse transcriptase inhibitor (NNRTI), a boosted protease inhibitor (PI), or an integrase strand transfer inhibitor (INSTI),^[1]^ has enabled control of HIV-1 infection with efficacy rates above 90% in recent clinical trials,^[2]^ progressive restoration of the immune system, and therefore, a significant reduction in acquired immunodeficiency syndrome (AIDS) events and other complications associated with HIV-1 infection itself. Restoration of the immune system was defined as an increase in the CD4+ T-cell count to normal values and an improved CD4/CD8 ratio >0.9.^[3]^

Despite improvements in triple therapy in terms of potency, efficacy, ease of dosing with single-tablet-a-day regimens,^[4]^ and better tolerability and safety, the currently used NRTI backbones are not entirely free of potential tolerability and toxicity issues.^[5,6]^ The increased potency of new third drugs, mainly INSTIs, together with the persistent toxicity of NRTIs, has led to the study and approval of therapeutic strategies based on 2 drugs (dual therapy), which makes it possible to avoid classic NRTIs.^[7]^ These combinations have been approved for use in both naïve and experienced patients.^[8]^ They guarantee not only undetectability, but also better tolerability and safety profiles, with less risk of developing potential short or long-term toxicities, especially in patients with comorbidities. Moreover, adherence may improve in some patients, and the costs are reduced.

Dolutegravir (DTG) is a highly potent and effective INSTI that has become the gold standard treatment for naïve patients.^[9–11]^ Dual therapy with DTG and lamivudine (3TC) or rilpivirine (RPV) has been approved for treatment-naïve (DTG/3TC) and experienced patients (DTG/3TC and DTG/RPV). The combination of DTG plus RPV was studied as a switching strategy in SWORD-1 and SWORD-2 studies and small real-world cohorts, which showed very favorable efficacy, safety, and tolerability.^[12–16]^ Data regarding the impact of dual therapy on CD4+ and CD8+ T lymphocyte counts and CD4/CD8 ratio in treatment-experienced patients are scarce. CD8+ T-cell count and CD4/CD8 ratio are indirect markers of immune activation and inflammation.^[17–20]^ Improvement in these laboratory markers with dual therapy is controversial and uncertain because data are scarce, and some clinicians doubt that dual therapy is as potent as triple therapy in restoring the immune system and reducing immune activation.

This study aimed to provide data on the impact of this strategy in real-life patients regarding changes in immune status. We explored efficacy and immune status based on CD4+ and CD8+ T lymphocyte counts in a cohort of 524 patients from 34 hospitals in Spain.

## 2 Material and methods

### 2.1 Patients and study design

**Figure 1:**
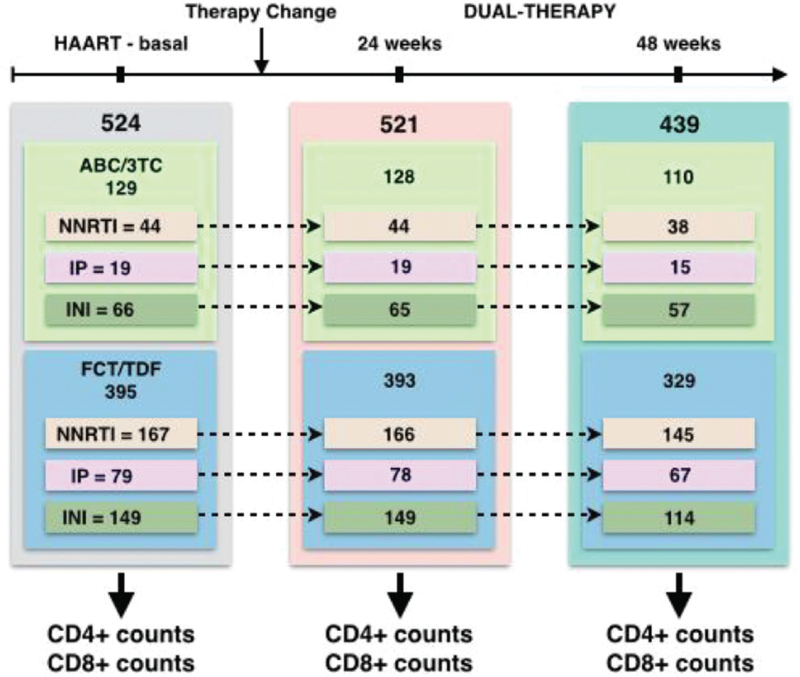
Experimental design. Treatment stages and samples sizes. Two different backbone drugs (ABC/3TC and FCT/TDF) and three different third agents (NNRTI, IP, and INI) were used in the HAART-baseline stage of the treatment. Patients were followed 24 and 48 weeks after the treatment change to the dual-therapy. HAART = highly active antiretroviral therapy, ABC/3TC = Abacavir/lamivudine, FCT/3TC = Tenofovir/emtricitabine, IP = Protease inhibitor, INI = Integrase strand transfer inhibitor, NNRTI = non-nucleoside reverse transcriptase inhibitor.

We performed a retrospective study of 524 virologically suppressed HIV-1-infected patients who switched to dual therapy with DTG plus RPV in 34 hospitals across Spain from June 2018 to May 2019. A systematic search of the databases of each hospital was performed to retrospectively select the appropriate candidates. All included patients fulfilled the following criteria: a) HIV-1 infection with age ≥18 years, b) switching from three-drug combination antiretroviral treatment (cART) to dual therapy with DTG+RPV, c) HIV RNA viral load <50 copies/ml in the previous 24 weeks before switching, and d) switching to DTG plus RPV at least 48 weeks before the start of the study in May 2020. Data were collected from medical records, anonymized, and entered into an online electronic database, REDCap^[21]^ (Fig. 1). The data collected included demographics (age, sex, and race), HIV-related data (route of HIV transmission, CD4 nadir, first viral load, AIDS stage, hepatitis co-infections, previous virological failures, antiretroviral regimens before switching, reasons for switching), pre-existing comorbidities, tolerability, safety profiles, and laboratory results.

The study protocol was approved by the Ethics Committee of Complejo Hospitalario de Avila in August 2020 (code 3/20) and was re-approved or registered by other local ethics committees. Given the retrospective nature of the study and the fact that data were obtained from routine clinical records, written informed consent was not necessary.

### 2.2 Outcomes

The primary outcome was efficacy analysis to determine the proportion of patients with undetectable viral loads (<50 copies/ml) at weeks 24 and 48. Secondary outcomes included the following: a) changes in the immune status of patients in terms of CD4+ and CD8+ T lymphocyte counts (cell/mm^3^) and CD4+/CD8+ ratio, b) safety profiles at weeks 24 and 48, and c) reasons for switching to DTG+RPV.

The efficacy analysis at weeks 24 and 48 included virological failures, treatment changes secondary to tolerability or safety issues, dropouts, and physician decisions.

CD4+ and CD8+ T lymphocyte counts were obtained from 524 patients at different stages of treatment:

1. baseline cART with two different backbone drugs (ABC/3TC and emtricitabine/TDF) and three different third agents (NNRTI, PI, and INSTI);
2. at 24 weeks after switching to dual therapy with DTG+RPV; and
3. at 48 weeks after switching to dual therapy with DTG plus RPV (Fig. 1).

Given that the experimental design requires a study to compare CD4+ and CD8+ T lymphocyte counts in individuals at different stages, these data points must be considered paired. A series of variables that can interact with lymphocyte count was also obtained, including sex, age, and previous antiretroviral treatment. We also recorded a diagnosis of AIDS and a CD4 nadir below 200 cells/mm^3^.

### 2.3 Statistical analysis

The statistical methods applied in this study were specific to the paired data. Our main objective was to assess differences in the lymphocyte counts of individuals receiving cART at baseline versus 24 and 48 weeks after the administration of dual therapy. Thus, we started by applying a simple paired sample *t-test* or paired *t-test* with n/2–1 degrees of freedom. Using this approach, we tested the differences in lymphocyte counts between the groups (baseline cART vs 24 weeks dual therapy, baseline cART vs 48 weeks dual therapy, and 24 weeks dual therapy vs. 48 weeks dual therapy) without correcting for covariates or considering the effect of the backbone and third agents.

To include covariates and address the effect of the drugs used at baseline, we applied a multiple generalized linear mixed model. By considering the individual as a random effect, this model enabled us to address the paired nature of the data:


$$Y_{lymphocyte\;counts}=(\beta_{0}+b_{p,0p})+\beta_{1}X_{i}+\beta_{2}X_{j}+\beta_{3}X_{k}+\beta_{4}X_{l}+\beta_{5}X_{m}+e_{pijklm}$$

where b_p,0p_ is the random effect of each individual, β_1_X_i_ is the fixed effect of the different treatments to be tested (baseline cART, dual therapy after 24 weeks, and dual therapy after 48 weeks), β_2_X_j_ is the fixed effect of the backbone drug, β_3_X_k_ is the fixed effect of the third agent, β_4_X_l_ is the fixed effect of sex, and β_4_X_m_ is the fixed effect of age.

## 3 Results

The study population was comprised of 524 patients from 34 centers in Spain. The baseline characteristics are summarized in Table 1. Men accounted for 76.9% of the population, with a median age of 53 years (range:43–58 years). Active hepatitis co-infections were present in 71 patients (13.5%), of whom three presented with hepatitis B virus and 68 with hepatitis C virus (HCV) infections. The sexual transmission pathway was responsible for 58.4% of HIV infections, followed by 26.6% due to intravenous drug injectors. The median time of HIV diagnosis was 26.01 years (20.01–30.4). The nadir CD4+ T-cell count was 241 (91.2–405.5), and 17.8% of the patients had been diagnosed with AIDS.

**Table 1:** Patient characteristics.

Demographic	
Age *median (25%–75%)*	53 (43–58)
Male sex n (%)	395/513 (76.9%)
Spanish nationality n (%)	422/519 (81.3%)
Comorbidities n (%)	
Arterial hypertension	88/524 (16.7%)
Diabetes	41/524 (7.8%)
Dyslipidemia	135/524 (25.7%)
Heart disease	13/524 (2.5%)
Cerebrovascular disease	9/524 (1.7%)
Peripheral vascular disease	10/524 (1.9%)
Kidney failure	36/524 (6.9%)
Osteoporosis/Osteopenia	64/524 (12.2%)
Chronic pulmonary disease	36/524 (6.9%)
Psychiatric disorders	42/524 (8.1%)
Cancer	10/524 (1.9%)
Chronic liver disease	65/524 (12.4%)
HIV infection	
Transmission pathways n (%)	
Sexual intercourse	306 (58.4%)
Intravenous drug injectors	139 (26.6%)
Immune status median (25%*–*75%)	
Nadir CD4 (cells/mm^3^)	241 (91.2–405.5)
Baseline CD4 (cells/mm^3^)	702 (507.5–952.5)
Baseline CD8 (cells/mm^3^)	941.1 (633–1174)
Baseline CD4/CD8 ratio	0.85 (0.58–1.17)
AIDS diagnosis n (%)	93/519 (17.8%)
Time of diagnosis median (25%*–*75%)	
Global Cohort	26.01 (20.01–30.4)
AIDS patients	28.1 (20.4–31.1)
Non-AIDS patients	24.6 (19.0–29.0)
Previous treatment n (%)	
*Backbone*	
ABC/3TC	129/524 (24.6%)
FTC/TDF	395/524 (75.4%)
*Third Agent*	
PI	98/524 (18.7%)
INSTI	215/524 (41.0%)
NNRTI	211/524 (40.3%)
Reasons for switching n (%)	
Treatment simplification	338/524 (64.5%)
Toxicity	129/524 (24.7%)
Transition therapy to injectable drugs	24/524 (4.5%)
Drug Interaction	22/524 (4.2%)
Simplicity	10/524 (1.9%)
Cost	1/524 (0.2%)
Coinfections n (%)	
HBV diagnosis	114/512 (22.3%)
- HBsAg positive	3/114 (2.6%)
HCV positive ELISA	129/510 (25.3%)
HCV positive PCR	68/232 (29.3%)

Denominator indicates number of patients with available data. 3TC = lamivudine, ABC = abacavir, ELISA = enzyme-linked Immunosorbent assay, FTC = emtricitabine, HBsAg: surface antigen hepatitis B, HBV = hepatitis B virus, HCV = hepatitis C virus, INSTI = integrase strand transfer inhibitor, NNRTI = non-nucleoside reverse transcriptase inhibitor, PCR = polymerase chain reaction, PI = protease inhibitor, TDF = tenofovir disoproxil fumarate.

The percentages of patients with undetectable HIV viral load who reached weeks 24 and 48 with this switching strategy were 99.4% and 83.8%, respectively. Virological failure was recorded in only 3 patients (0.57%). Resistance mutations were not detected. No differences were found in terms of efficacy between the AIDS and non-AIDS subgroups using the general linear model (GLM): –0.536, *P* = .1803 or Fisher exact test: OR = 0.6301 (0.28–1.5); *P* = .279. At week 48, discontinuations due to reasons other than virologic accounted for 16.2% of cases, with 2.3% due to toxicity issues.

**Figure 2:**
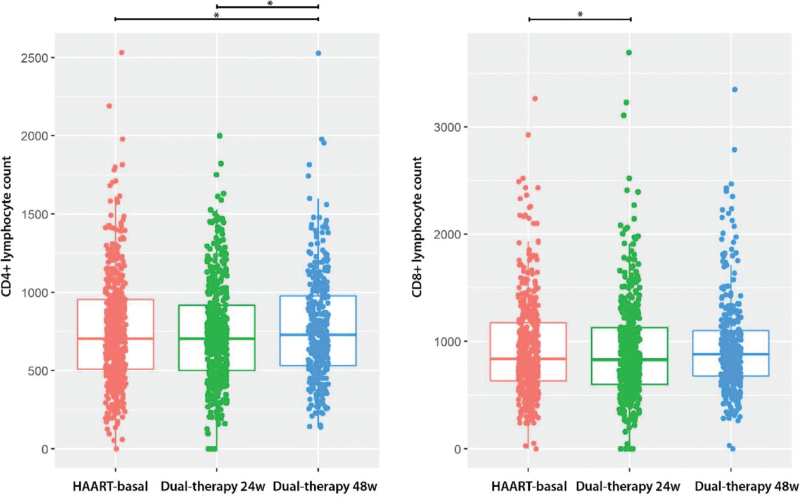
Box plot of CD4+ and CD8+ lymphocyte count. Lymphocyte count is shown for the three treatment stages: HAART-baseline treatment, 24 and 48 weeks after changing to a dual-treatment. Asterisks represent statistical significance under a t-test for paired data.

**Figure 3:**
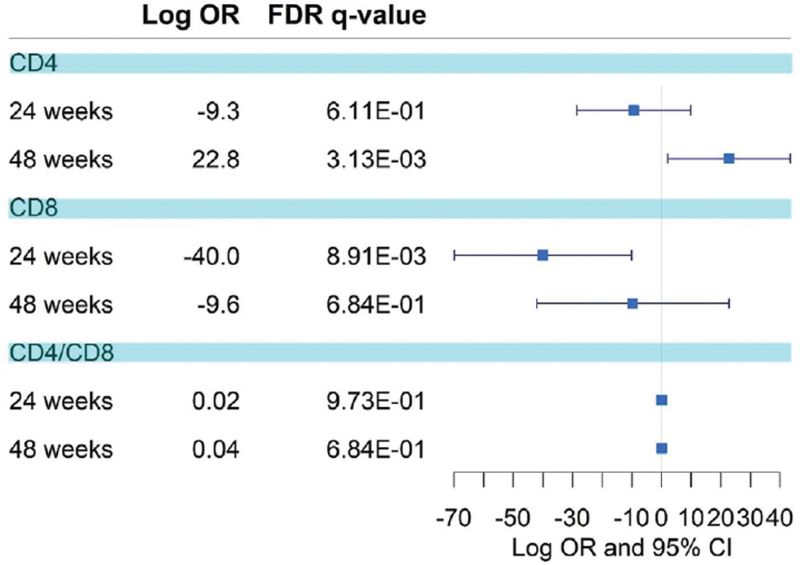
Forest plot of the treatment effect. Logarithm of the odds ratios, along with the 95% confident interval, for the treatment effect in the multiple linear mixed models are shown. Results are shown for CD4+ and CD8+ lymphocyte count, along with the ration CD4+/CD8+.

Immune status, assessed through the median baseline CD4+ and CD8+ T lymphocyte counts, was shown to change in the complete dataset. A simple analysis of paired data showed an increase in CD4+ T-cells (mean difference = 25.06, 95% CI = 3.11–47.01) at week 48 after switching treatment to dual therapy, and a decrease in CD8+ T-cells (mean difference = –35.9, 95% CI = –68.54 to –3.41) at week 24 after switching (Fig. 2, Supplementary Digital Content Table S1, Supplementary Digital Content Figures S1 and S2). Multiple mixed models considering the individual as a random effect and accounting for covariates such as sex and age corroborated this effect: subjects experienced a reduction in CD8+ T-cells at 24 weeks after switching (log OR = –40) and an increase in CD4+ T-cell count at 48 weeks (log OR = 22.82) (Fig. 3, Supplementary Digital Content Table S2). No significant changes were observed in the CD4+/CD8+ ratio (baseline = 0.849, 24 weeks = 0.87, and 48 weeks = 0.840). No significant differences were observed in the effect on the progression of the immune status of the baseline backbone or the third drug (NNRTI, INSTI, or boosted PI), sex, age >50 years, or the presence of active HCV co-infection in people who inject drugs (Welch two-sample t-test: effect = –0.054 (–0.27, 0.1615); *P* = .6177).

### 3.1 AIDS subgroup

AIDS was detected in 93 individuals from our cohort, of whom 66 were men, with a median age of 55 years (range, 47–62 years). Patients diagnosed with AIDS also showed a statistically significant increase in CD4+ T-cell counts. The mean difference between baseline cART and 48 weeks after switching was 46.34 (95% CI = 90.5–2.12) (Supplementary Digital Content Table S1). This effect was also significant in the multiple mixed models at 48 weeks (log OR = 41.78). No differences were found in the CD8+ T-cell count, effect of the baseline antiretroviral backbone, or third drug.

Adverse events (AEs) were reported in 20 patients (3.8%), including renal toxicity in 6 patients (35%), central nervous system toxicity in 6 (30%), and gastrointestinal issues in 4 (20%). No severe AEs were observed. Twelve patients (2.3%) discontinued treatment because of mild-to-moderate toxicity issues.

Changes in laboratory values included an increase in creatinine and estimated glomerular filtration rate (eGFR) at weeks 24 and 48 (week 24: creatinine log odds ratio [OR] = 0.0767, *P* = 6.47E-06; eGFR log OR = –4.37, *P* = 1.17E-10. Week 48: creatinine: log OR = 0.069, *P* = 1.42E-04 and eGFR log OR = –3.79; *P* = 1.85E-07). We did not find significant differences in the subgroup of 395 patients who used tenofovir disoproxil fumarate (TDF) as part of their previous treatment, apart from those who also used boosted PIs (log OR = 5.51, *P* = 3.4E-03).

The main reasons for switching to DTG plus RPV included simplification (64.5%) and toxicity of baseline cART (24.6%). Clinicians indicated this dual therapy with DTG plus RPV as a transition to future injectable treatments with RPV in 4.5% of the patients.

## 4 Discussion

The efficacy rates of the DTG plus RPV strategy are high in clinical trials and real-life cohort studies.^[12–16]^ In our cohort, these rates align with published data, around 100% at week 24, with only three virological failures due to poor adherence during the first 48 weeks, even in patients diagnosed with AIDS and those treated with multiple regimens. Similar results have been reported in previous real-life cohorts of patients undergoing dual therapy.^[16,22]^ These high rates of virological response make this regimen a valid alternative to triple therapy in virologically controlled HIV-1-infected patients without NNRTI resistance mutations, even in those with long-term disease, multiple previous treatments, and a prior diagnosis of AIDS.

Immune activation and biomarkers of inflammation improve within the first year after cART-induced HIV-1 suppression; however, some residual immune activation is present,^[3,18,19]^ and CD8+ T-cell count and, more specifically, the CD4/CD8 ratio, are indirect markers of immune activation and inflammation, both of which affect prognosis. Some retrospective and observational cohort studies have suggested that dual therapy could lead to less powerful control of HIV replication, with a subsequent negative impact on immune status in the form of increased activation, inflammation, and lower CD4/CD8 ratios.^[17,19]^ In our cohort, no significant changes were observed in the CD4/CD8 ratio (0.85 at baseline) during the first 48 weeks after switching to DTG plus RPV. Similar findings have been reported in patients diagnosed with AIDS. Our data contrast with other published real-life cohort studies, which reported a slight increase in the CD4/CD8 ratio,^[22]^ possibly because of the lower baseline CD4/CD8 ratio (0.71) and smaller study population (91 patients in the DTG/RPV group).

Some authors reported an increase in the CD8+ T-cell count after switching 104 patients to dual therapy with boosted PIs.^[23]^ In our cohort of 524 patients, we observed a significant decrease in the CD8+ T-cell count at week 24 and an increase in the CD4+ T-cell count at week 48, with a switching baseline count >700 cells/mm^3^. It is mandatory to note this aspect in our cohort, because, despite a general low nadir of CD4+ T-cell count or a long-term HIV infection, the CD4+ T-cell count at the switching time was over 500 cells/mm^3^, and the CD4/CD8 ratio was 0.85, in contrast with published data. This suggests that most patients fail to normalize their ratio if they start treatment during chronic HIV infection, even after a decade of viral suppression.^[24]^

Our findings suggest that in treatment-experienced patients with virologically controlled HIV infection and a high CD4+ T-cell count, especially those with a long history of HIV infection, more than 48 weeks are needed to evaluate the significant changes in the CD4/CD8 ratio after switching.^[25]^ Moreover, the impact of dual therapy on the restoration of the immune system could differ depending on the antiretrovirals used, including INSTIs, boosted PIs,^[23]^ and NNRTIs. Nevertheless, long-term follow-up would probably be necessary to account for changes in immune status with DTG plus RPV.

Co-infection with other viruses may play an important role in immune activation. In our cohort, we found no difference in patients with active HCV infection, but we were unable to determine the potential impact of other viruses, such as cytomegalovirus due to the retrospective nature of the study.

The DTG plus RPV strategy was well-tolerated and safe, with AEs registered in only 3.8% of the patients. These results correlate with published data from clinical trials and cohort studies.^[12,16]^ This observation is of great interest because toxicity continues to be one of the main reasons for switching antiretrovirals,^[26]^ although only 2.3% of patients in our cohort discontinued treatment due to mild or moderate issues. The main reasons for withdrawal were physicians’ decisions or clinical conditions other than HIV infection, which prevented continuation of DTG plus RPV. Good safety and tolerability profiles make this regimen a simplified option for patients with significant comorbidities who need drugs that are readily metabolized and for patients who have or are predisposed to toxicities.

In our cohort, no improvement in renal function (creatinine or eGFR) was observed in patients whose previous regimen included TDF except when boosted PIs were present. Remarkably, the combination of boosted PIs and TDF is associated with renal problems.^[27]^ This result contrasts with data from clinical trials and real-life cohort studies,^[12,16,22]^ which found significant changes in renal function after discontinuing TDF. This may be explained by chronic damage to renal function in patients with long-term exposure to TDF. DTG plus RPV have a good renal profile and constitute a favorable option for this group of patients.

Our study was limited by its retrospective design and the absence of a control group. In addition, the clinical protocols and visit timetables differed between the participating hospitals. Nevertheless, the strength of this study is its large sample size (>500 patients), which is greater than that of other real-life cohorts (approximately ≤100 patients). Further investigations are needed to elucidate this aspect in clinical trials and prospective observational studies.

In conclusion, administration of dual therapy with DTG plus RPV to treatment-experienced and virologically controlled patients was highly effective and slightly improved the immune status during the first 48 weeks after switching, in terms of not only the CD4+ T-cell count but also the CD8+ T-cell count. The low tolerability and safety issues, together with the low number of discontinuations owing to these toxicity issues, make this regimen a good switching strategy for aging patients, those with several comorbidities, and those with previous or potential long-term toxicities.

## Acknowledgments

We thank the patients, investigators, and participating institutions for enabling this study possible.

## Author contributions

**Conceptualization:** Carlos Dueñas, Jesús Troya, Luis Buzón.

**Data curation:** Carlos Dueñas, Jesús Troya, Luis Buzón.

**Formal analysis:** Francisco Ceballos, Jesús Troya.

**Investigation:** Ana Pelaez, Elisabeth Delgado, Jesús Troya, Luis Buzón, María Garcinuño, María José Galindo, Marta Montero, Miguel Ángel Morán, Miguel Cervero, Sara de la Fuente, Teresa Aldamiz, Carlos Dueñas, Guillermo Posada, Idoia Irazola, Ignacio Santos, Javier Fuente, Josean Uribaren, Josefa Galindo, Julia Gomez.

**Methodology:** Carlos Dueñas, Jesús Troya, Luis Buzón.

**Project administration:** Jesús Troya.

**Supervision:** Jesús Troya.

**Writing – original draft:** Carlos Dueñas, Luis Buzón, Jesús Troya.

**Writing – review & editing:** Ana Pelaez, Luis Buzón, Carlos Dueñas, Carlos Galera, Cristina Hernández, Desiré Gil, Elisabeth Delgado, Estela Moreno-García, Francisco Ceballos, Guillermo Posada, Idoia Irazola, Ignacio Santos, Javier Fuente, Jesús Troya, Jose Antonio Iribarren, Jose Manuel Guerra, Josean Uribaren, Josefa Galindo, Julia Gomez, María Garcinuño, María José Galindo, Marta Montero, Miguel Ángel Morán, Miguel Cervero, Sara de la Fuente, Teresa Aldamiz.

## Supplementary Material

Supplemental Digital Content

## Supplementary Material

Supplemental Digital Content

## Supplementary Material

Supplemental Digital Content
