# Supplementary material for: Dolutegravir plus rilpivirine: benefits beyond viral suppression: DORIPEX retrospective study
Source: Medicine (Baltimore). 2022 Jun 17;101(24):e29252. doi: 10.1097/MD.0000000000029252 (PMC9276328; doi:10.1097/MD.0000000000029252)
Supplement: Supplemental Digital Content [file medi-101-e29252-s001.docx]

**Supplementary figure 1**. **Differences in CD4+ and CD8+ lymphocyte counts between treatments.**


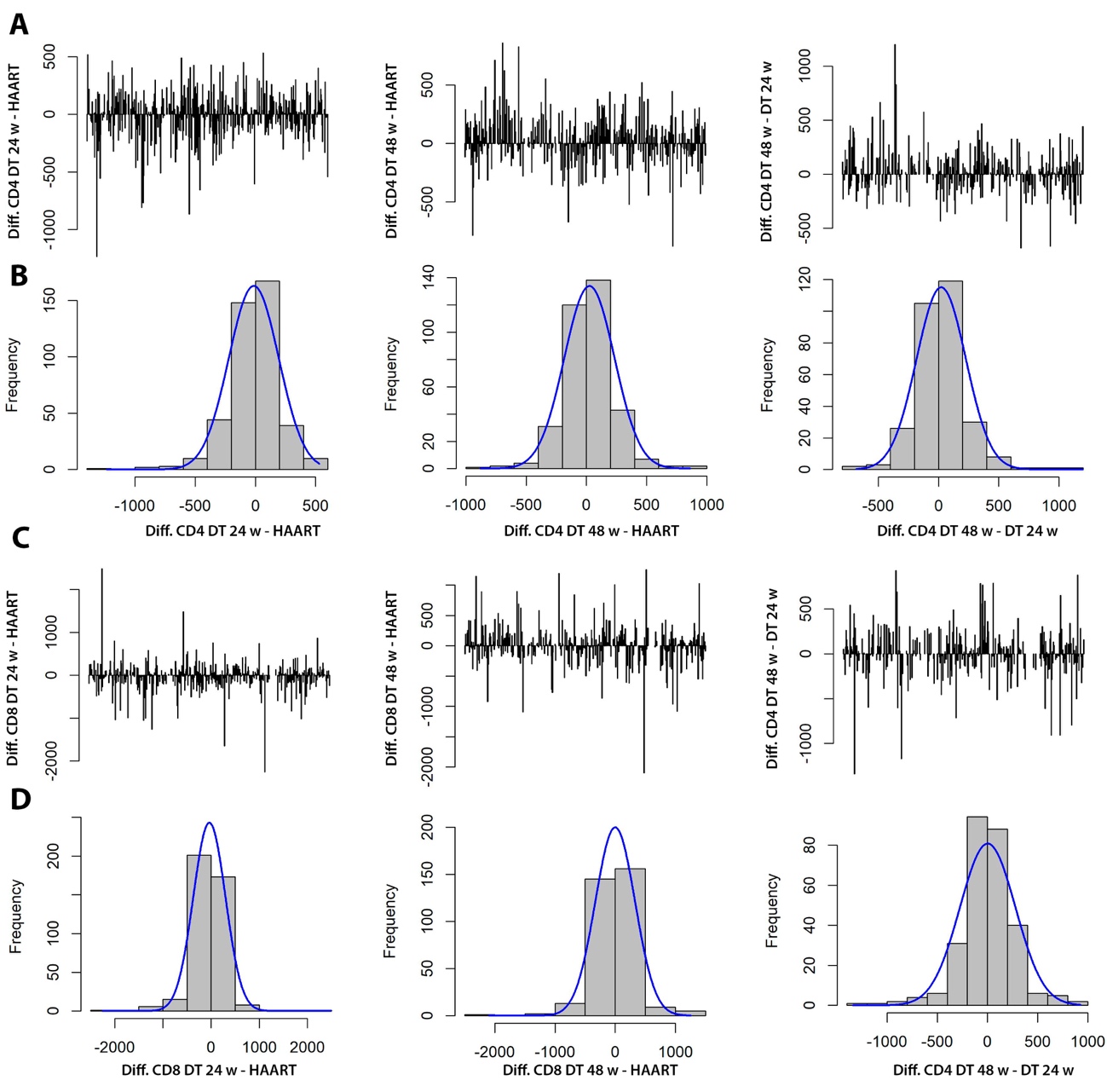


**A)** Pairwise differences between CD4+ counts in the same individual at different stages of treatment (baseline HAART vs. 24 weeks after switching to dual therapy, baseline HAART vs. 48 weeks after switching to dual therapy and 24 weeks after switching to dual therapy vs 48 weeks after switching to dual therapy. **B)** Histogram and curve estimation of differences between CD4+ counts on the same individual at different stages of treatment (baseline HAART vs. 24 weeks after switching to dual therapy, baseline HAART vs. 48 weeks after switching to dual therapy and 24 weeks after switching to dual therapy vs 48 weeks after switching to dual therapy. **C)** Pairwise differences between CD8+ counts on the same individual at different stages of the treatment (baseline HAART vs. 24 weeks after switching to dual therapy, baseline HAART vs. 48 weeks after switching to dual therapy and 24 weeks after switching to dual therapy vs 48 weeks after switching to dual therapy. **D)** Histogram and curve for the estimation of differences between CD8+ counts in the same individual at different stages of treatment (baseline HAART vs. 24 weeks after switching to dual therapy, baseline HAART vs. 48 weeks after switching to dual therapy and 24 weeks after switching to dual therapy vs. 48 weeks after switching to dual therapy.
