# Supplementary material for: Dolutegravir plus rilpivirine: benefits beyond viral suppression: DORIPEX retrospective study
Source: Medicine (Baltimore). 2022 Jun 17;101(24):e29252. doi: 10.1097/MD.0000000000029252 (PMC9276328; doi:10.1097/MD.0000000000029252)
Supplement: Supplemental Digital Content [file medi-101-e29252-s002.docx]

**Supplementary table 1. t Test for paired data.**

|  | **Estimate** | **CI** | | **P-value** |
| --- | --- | --- | --- | --- |
| **Complete cohort** | |  |  |  |
| *CD4* |  |  |  |  |
| Baseline vs 24 | -14.1 | -33.96 | 5.71 | 1.62E-01 |
| Baseline vs 48 | 25.06 | 3.11 | 47.01 | 2.53E-03 |
| *CD8* |  |  |  |  |
| Baseline vs 24 | -35.97 | -68.53 | -3.41 | 3.04E-03 |
| Baseline vs 48 | -1.18 | -36.88 | 34.52 | 9.48E-01 |
| *CD4/CD8* |  |  |  |  |
| Baseline vs 24 | 0.049 | -0.166 | 0.264 | 6.54E-01 |
| Baseline vs 48 | 0.007 | -0.0622 | 0.0774 | 8.30E-01 |
| **AIDS-diagnosed patients** | |  |  |  |
| *CD4* |  |  |  |  |
| Baseline vs 24 | -3.706 | -38.54 | 31.13 | 8.33E-01 |
| Baseline vs 48 | 46.34 | 12.105 | 80.584 | 4.30E-03 |
| *CD8* |  |  |  |  |
| Baseline vs 24 | -61.64 | -135.95 | 12.67 | 1.03E-01 |
| Baseline vs 48 | -9.54 | -91.23 | 72.15 | 8.16E-01 |
| *CD4/CD8* |  |  |  |  |
| Baseline vs 24 | 0.009 | -0.266 | 0.364 | 8.54E-01 |
| Baseline vs 48 | 0.012 | -0.1622 | 0.1774 | 7.88E-01 |

Estimated mean difference, 95% confidence intervals, and P-values are provided for the analysis of the complete cohort and AIDS-diagnosed patients.
