# Supplementary material for: Dolutegravir plus rilpivirine: benefits beyond viral suppression: DORIPEX retrospective study
Source: Medicine (Baltimore). 2022 Jun 17;101(24):e29252. doi: 10.1097/MD.0000000000029252 (PMC9276328; doi:10.1097/MD.0000000000029252)
Supplement: Supplemental Digital Content [file medi-101-e29252-s003.docx]

**Supplementary table 2. Multiple linear mixed model outcomes.**

|  | CD8+ | | | CD4+ | | | | CD4+/CD8+ | | | | | |  |  |
| --- | --- | --- | --- | --- | --- | --- | --- | --- | --- | --- | --- | --- | --- | --- | --- |
|  | Estimate | P-value | | Estimate | | P-value | | Estimate | | | P-value | | |  |  |
| **Demographic variables** | | | | |  | |  | |  | | |  | | |  |
| *Complete cohort* | | |  | |  | |  | |  | | |  | | |  |
| Sex | 121.42 | 6.81E-03 | | -30.87 | | 3.49E-01 | | -0.29 | | | 2.72E-03 | | |  |  |
| Age | -2.27 | 1.63E-01 | | -5.32 | | 1.04E-05 | | 0.00 | | | 8.00E-01 | | |  |  |
| *AIDS-diagnosed patients* | | |  | |  | |  | |  | | |  | | |  |
| Sex | 49.70 | 6.71E-01 | | -77.49 | | 3.19E-01 | | 0.40 | | | 2.84E-02 | | |  |  |
| Age | -2.97 | 5.37E-01 | | 0.68 | | 8.31E-01 | | 0.00 | | | 8.43E-01 | | |  |  |
| **Backbone drug as fixed effect: ABC/3TC as baseline** | | | | | | | | | |  | | |  | | |
| *Complete cohort* | | |  | |  | |  | |  | | |  | | |  |
| FCT/TDF | -50.17 | 2.56E-01 | | -38.87 | | 2.34E-01 | | 0.01 | | | 8.94E-01 | | |  |  |
| *AIDS-diagnosed patients* | | |  | |  | |  | |  | | |  | | |  |
| FCT/TDF | -57.23 | 6.47E-01 | | -131.49 | | 1.11E-01 | | 0.14 | | | 5.60E-01 | | |  |  |
| **Third agent as fixed effect: PI as baseline** | | | | | | |  | |  | | |  | | |  |
| *Complete cohort* | | |  | |  | |  | |  | | |  | | |  |
| INI | 47.00 | 3.77E-01 | | -18.59 | | 6.28E-01 | | -0.09 | | | 4.22E-01 | | |  |  |
| NNRTI | -28.61 | 4.92E-01 | | 29.88 | | 1.21E-01 | | 0.12 | | | 1.82E-01 | | |  |  |
| *AIDS-diagnosed patients* | | |  | |  | |  | |  | | |  | | |  |
| INSTI | 96.17 | 4.68E-01 | | 46.35 | | 5.98E-01 | | -0.10 | | | 6.71E-01 | | |  |  |
| NNRTI | -196.18 | 1.15E-01 | | 125.16 | | 1.30E-01 | | 0.24 | | | 2.96E-01 | | |  |  |
| **Treatment fixed effect: HAART at baseline** | | | | | | |  | |  | | |  | | |  |
| *Complete cohort* | | |  | |  | |  | |  | | |  | | |  |
| Dual-therapy after 24 weeks | -40.00 | 8.91E-03 | | -9.36 | | 6.11E-01 | | 0.02 | | | 9.73E-01 | | |  |  |
| Dual-therapy after 48 weeks | -9.67 | 6.84E-01 | | 22.82 | | 3.13E-03 | | 0.04 | | | 6.84E-01 | | |  |  |
| *AIDS-diagnosed patients* | | |  | |  | |  | |  | | |  | | |  |
| Dual-therapy after 24 weeks | -41.07 | 2.65E-01 | | 0.11 | | 9.95E-01 | | -0.08 | | | 4.44E-01 | | |  |  |
| Dual-therapy after 48 weeks | -23.49 | 5.48E-01 | | 41.78 | | 3.80E-03 | | -0.10 | | | 3.33E-01 | | |  |  |

Estimates and P-values are provided for the analysis of the complete cohort and AIDS-diagnosed patients.
